# Public mental health stigma and suicide rates across Europe

**DOI:** 10.3389/fpubh.2025.1554072

**Published:** 2025-06-03

**Authors:** Lara Oblak

**Affiliations:** Mind & Brain Lab, Department of Psychology, Faculty of Arts, University of Ljubljana, Ljubljana, Slovenia

**Keywords:** mental health stigma, suicide, stigma measurement, public health, Eurobarometer

## Abstract

**Introduction:**

Mental health stigma remains a significant public health concern, particularly due to its complex relationship with suicide risk. While the two phenomena appear to be closely connected, inconsistencies in stigma measurement and a lack of standardized instruments complicate efforts to fully understand its role in suicide occurrence and prevention.

**Methods:**

We analyzed stigma measures from the 2022 and 2023 Eurobarometer surveys, alongside suicide rates and socio-economic indicators for 27 European Union countries. Correlational analyses and hierarchical linear regression models were employed to assess the relationships between stigma-associated variables and national suicide rates.

**Results:**

Our analysis revealed a notable decline in suicide rates between 2010 and 2019, with only four countries reporting increases. We found multiple negative associations between suicide rates and stigma measures, notably for the belief that disclosing a mental health condition would negatively impact one's career. Hierarchical linear regression models supported this item as a significant predictor of lower suicide rates.

**Discussion:**

The findings underscore the need for more systematic, theory-driven approaches to stigma assessment, as inconsistencies in survey items and temporal mismatches between stigma and suicide data hinder efforts to draw conclusive inferences. By employing robust measurement tools and systematic surveillance of mental health attitudes on a multinational scale, future research can better illuminate the complex interplay between stigma and suicidal behavior, ultimately enhancing our efforts toward suicide prevention.

## 1 Introduction

Suicidal behavior is a complex and pervasive issue, inflicting dire consequences on those most intimately involved with it, and presenting a major concern to global public health. According to most recent reports of the World Health Organization, the annual global death toll of suicide in 2019 was over 700,000, with suicide attempts greatly exceeding this number. In the same year, suicide was the fourth leading cause of death among 15–29-year-olds globally (1).

The widespread occurrence of suicide is inextricably linked to the issue of stigmatization, which continues to shape public perceptions of mental health and suicide prevention (2). Stigma is a multifaceted concept describing negative attitudes and behaviors toward a specific group (3), which manifests on a societal, interpersonal, and individual level. While multiple conceptual frameworks of stigmatization have been proposed, most distinguish between public and self-stigma. Public stigma encompasses societal beliefs and reactions to individuals perceived to have a stigmatized condition, whereas self-stigma occurs when individuals who belong to a stigmatized group apply and internalize negative perceptions held by the public (4, 5).

In relation to those affected by mental health conditions, stigmatization often includes perceiving these individuals as dangerous, weak, or responsible for their own condition, and is frequently related to feelings of fear, anger and pity (6). When these notions are perceived or internalized by stigmatized individuals, they pose a significant risk factor for suicide, as they may result in profound distress, diminish self-esteem, and obstruct efforts to seek help (2). In addition to stigmatization of mental illness itself, suicide stigma tends to label suicide attempt survivors as cowardly, selfish, or failures, while those grieving a loved one's suicide are often perceived as guilty, broken, or pitiable, which is related to greater distress and increased suicidality among both groups (7). While suicide stigma and mental illness stigma are closely related, suicide survivors seem to experience additional, suicide-specific stigmatization, such as being labeled as selfish or immoral (7, 8). The present work focuses specifically on public stigmatizing attitudes toward mental illness in general, rather than stigma associated with suicide, and investigates how these societal attitudes relate to national suicide rates across European countries.

There is considerable variability in the levels of suicide and mental illness stigma across different countries and regions (9). This diversity likely arises from a complex interplay of many factors, among them cultural attitudes, socio-economic conditions and religious beliefs. Notably, differing public views toward suicide and mental health issues may contribute significantly to this inter- and cross-national variability in suicide rates. One of the first works examining this relationship compared two neighboring regions in Europe, and found higher levels of self-stigma, shame and aversion to help seeking in Flanders, a region with a significantly higher suicide rate as compared to its neighboring region of Holland (10). These conclusions pointed to the potential role of stigmatization in explaining the differences in suicide rates across various countries and cultures. In 2015, Schomerus et al. expanded on these findings by comparing levels of stigmatization and suicidal behavior across 25 European countries (11). They combined country-level data on social acceptance from a Flash Eurobarometer survey with suicide rates and socio-economic indicators. Public stigma was quantified based on a survey item asking respondents whether they would feel uncomfortable talking to someone with a significant mental health problem. A higher level of social acceptance, indicated by fewer people expressing discomfort, was linked to lower age-standardized national suicide rates, even after controlling for socio-economic factors. This study highlighted the significant variation in stigma and its association with suicidal tendencies among different populations.

Since 2010, considerable efforts have been made to reduce stigma through public health campaigns and policy changes aimed at improving mental health outcomes and reducing suicide rates (12). This raises the question of whether the rates of stigmatization and suicidal behavior have indeed been altered in light of these efforts. In this work, we aim to assess the relationship between suicide rates and mental illness stigma a decade after the initial formal investigations into their interconnection. By examining most recent available data and trends, we hope to provide insights into the current state of this relationship, which we believe to be highly relevant both for assessing our efforts thus far as well as informing future strategies to combat stigma and further reduce the incidence of suicidal behavior.

## 2 Materials and methods

### 2.1 Stigma prevalence

To extract stigma-related measures, we reviewed data from two Eurobarometer surveys reporting on mental health. All sections and items from the two reports were screened. As the reports covered a wide range of health-related topics, only those items that were deemed to reflect the presence or perception of stigmatization were selected for inclusion in the analysis. This resulted in three sets of survey items used to assess stigma prevalence. The first two sets were collected from the Mental Health Eurobarometer survey conducted in June 2023 (13). Data was collected between 14 and 21 June 2023 on a population of European Union citizens aged 15 and over (*n* = 26,501). The collection process was carried out online by means of Computer-Assisted Web Interviewing (CAWI). As an indicator of stigma, we focused on Section 4 of the survey, which assessed perceptions about people with mental health issues. Specifically, we focused on items Q12 and Q13, both of which consisted of several questions.

The Q12 set of questions contained three sub-items (termed Q12_1, Q12_2 and Q12_3). Q12_1 asked participants the following: “Do you think that mental health patients are judged differently than other patients by society in general?” Items Q12_2 and Q12_3 posed an identical question, but asked whether participants believe mental health patients are judged differently by medical professionals or by people in educational or professional settings, respectively. Participants could respond with (1) Yes, (2) No, or (3) Don't know. For each of the items, the level of perceived stigmatization was computed as the proportion of participants who responded with “Yes,” indicating that mental health patients are judged differently than other patients. A higher proportion of “Yes” responses was thus taken as an indication of higher levels of perceived stigmatization of mental health patients.

The Q13 set of items asked participants “To what extent do you agree or disagree with the following statements about people with mental health issues in [YOUR COUNTRY]?” Six statements were presented, corresponding to items Q13_1 through Q13_6: (1) People with mental health issues receive the same level of care as those with a physical condition, (2) Mental health promotion is as important as physical health promotion, (3) People with mental health issues are perceived as less capable and contributing less to society, (4) People with mental health issues are seen as less sociable, (5) People with mental health issues get less opportunities at work, in finding housing, in social activities etc., (6) Mental health issues are perceived as not curable. Participants could respond with (1) Totally agree, (2) Tend to agree, (3) Tend to disagree, (4) Totally disagree, or (5) Don't know. The level of perceived stigmatization was computed as the combined proportion of participants who responded with “Totally agree” and “Tend to agree,” yielding 6 scores of stigmatization.

A third set of measures of mental health stigmatization was taken from the Flash Eurobarometer “OSH Pulse—Occupational safety and health in post-pandemic workplaces” (14). Survey responses were collected between 25 April and 23 May 2022 via Computer-Assisted Telephone Interviewing (CATI). The target population were employed European Union citizens aged 16 and over (*n* = 25,683). The contacted telephone numbers were obtained through Random Digit Dialing (RDD) methods.

Following item screening, Section 3 of the report was chosen as it focused on mental health. Specifically, items from Subsection 3.1 (item E2_1 and E2_2) were included, as they addressed the perceived negative implications of discussing mental health issues within the workplace. In item E2_1, participants were asked whether they agree or disagree with the following statement: “Disclosing a mental health condition would have a negative impact on my career.” In item E2_2, they were asked for their level of agreement with the statement “I would feel comfortable speaking to my manager or supervisor about my mental health.” Participants could reply with (1) Strongly agree, (2) Agree, (3) Disagree, (4) Strongly disagree, or (5) Don't know. Cumulative percentages of “Strongly agree” and “Agree” responses were computed for each item and used as a proxy score of mental health stigmatization.

In both Eurobarometer datasets, we included data from all 27 EU member states: Austria, Belgium, Bulgaria, Croatia, Republic of Cyprus, Czech Republic, Denmark, Estonia, Finland, France, Germany, Greece, Hungary, Ireland, Italy, Latvia, Lithuania, Luxembourg, Malta, Netherlands, Poland, Portugal, Romania, Slovakia, Slovenia, Spain, and Sweden.

### 2.2 Suicide rates

Age-standardized suicide rates per 100,000 inhabitants were obtained from multiple sources to account for data availability limitations and provide a comprehensive analysis. As country-wide stigmatization data were only assessed in large international surveys conducted by the European Commission in 2010 and 2023, this constrained our analyses of the stigma-suicide relationship. Notably, suicide rate data for 2023 were unavailable at the time of this study, necessitating the use of estimates from earlier years to approximate this relationship. A multi-dataset approach was undertaken to examine the stigma-suicide association as accurately as possible given these data availability limitations.

Data for 2010 and 2019 were collected from the World Health Organization (WHO) Global Health Estimates (1). The WHO 2019 dataset was chosen as it aligns most closely with the dataset used by Schomerus et al. (11).

To incorporate more recent information, suicide rate estimates for 2021 were additionally sourced from Eurostat (15). Lastly, in an additional effort to supplement these datasets, we manually collected most recent available suicide rate information from the national statistics offices of each EU country. This process yielded estimates for 14 EU countries for 2023 and 7 countries for 2022. These data represent the most recent available figures at the time of the study.

### 2.3 Socio-economic indicators

As macroeconomic factors such as Gross Domestic Product (GDP) and levels of unemployment have been linked to suicide rates (16–18), we collected information on three socio-economic indicators for each of the countries included in the analysis. Specifically, we extracted data on GDP *per capita*, unemployment rates, and GINI coefficients of income inequality. Data were gathered for the year 2019, 2021, and 2023 from the *nama_10_pc, tessi190*, and *tipsun20* online datasets provided by Eurostat (19–21).

### 2.4 Statistical analysis

We conducted a systematic series of analyses to evaluate the relationship between suicide rates and stigmatization, employing data from three distinct datasets: the 2019 dataset (WHO suicide rates and socioeconomic indicators from 2019), the 2021 dataset (Eurostat suicide rates and socioeconomic indicators from 2021), and the 2022/23 dataset (manually gathered suicide rates from 2022/23 and socioeconomic indicators from 2023). Each dataset was analyzed individually to ensure consistency and account for variations in data availability. The analyses included pairwise correlations, focused correlation plots, and hierarchical linear regression modeling to identify predictors of suicide rates. All analyses were conducted using R software (22).

#### 2.4.1 Cross-year comparison of WHO suicide estimates

Changes in suicide rates over time were assessed using a paired *t-*test comparing suicide mortality in 2010 and 2019. A Pearson correlation analysis was conducted to assess the consistency of suicide rates across countries, evaluating whether higher rates in 2010 were associated with higher rates in 2021. Given the differences in data collection methodologies, we did not extend this comparison to Eurostat or manually collected suicide data for 2021 and 2022/23, as these datasets are not directly comparable with WHO estimates.

#### 2.4.2 Correlation analysis

Pairwise Pearson correlations were first computed within each dataset to explore potential interrelationships among socio-economic indicators, stigma measures, and suicide rates. These correlations were exploratory and did not directly inform linear model construction. To specifically examine associations with suicide, correlations of each stigma measure and socio-economic indicator with suicide rates were calculated for the 2019, 2021, and 2022/23 dataset. Within each dataset, the absolute values of these correlations were ranked in descending order and used to guide the construction of regression models based on their strength of association with suicide rates.

#### 2.4.3 Regression modeling

Based on the correlations with suicide rates, hierarchical linear regression models were constructed for each dataset to examine predictors of suicide rates. Initially, the variable most strongly correlated with suicide rates was used as a single predictor. Subsequent variables were added stepwise to the model, in order of descending correlation strength. Analysis of variance (ANOVA) was used to evaluate whether the inclusion of additional predictors significantly improved model fit. In each dataset, this stepwise process was repeated with the four variables which showed the highest absolute correlation values.

## 3 Results

**Figure 1:**
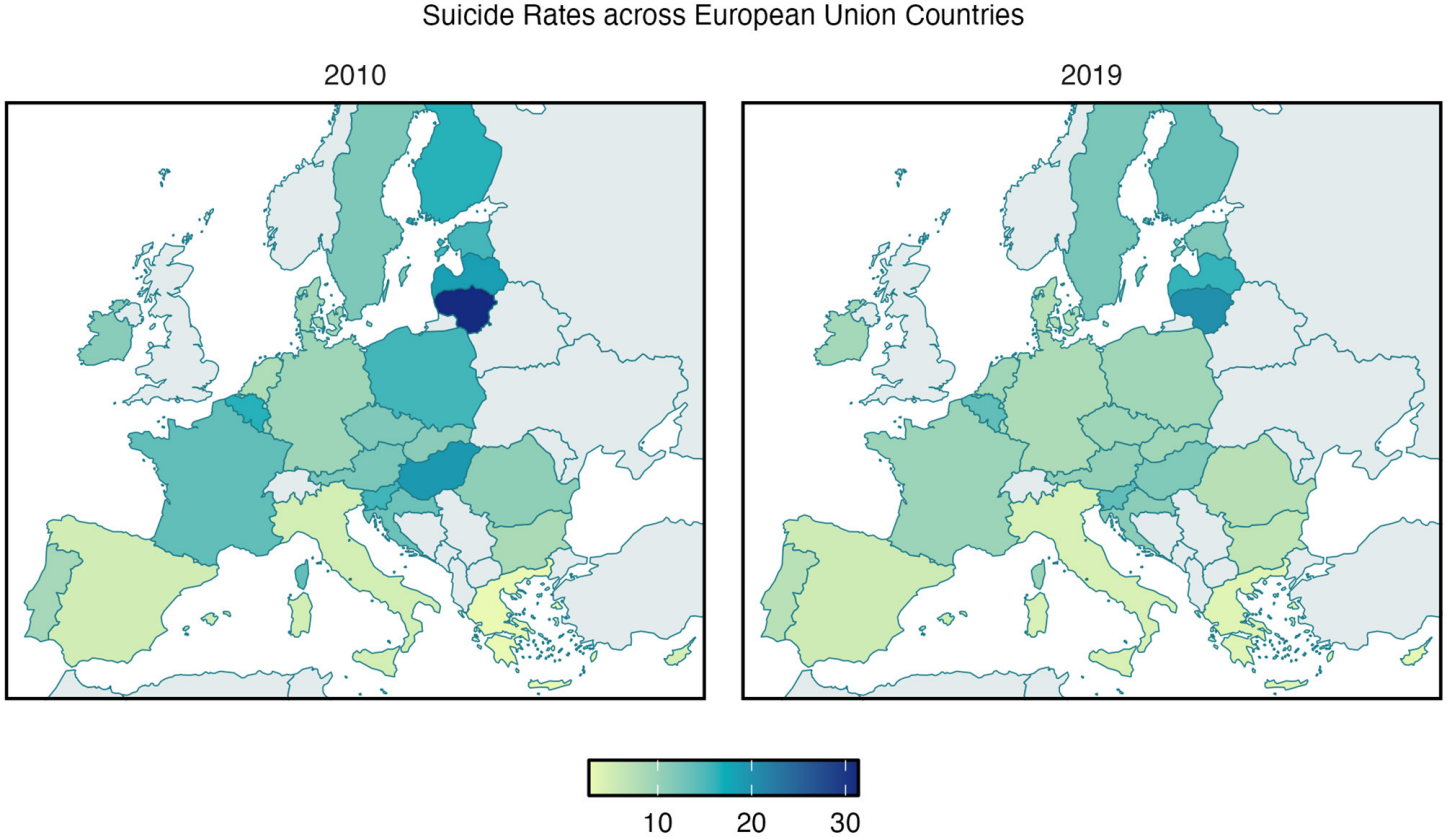
Age-standardized suicide rates in EU Member States in 2010 **(left panel)** and 2019 **(right panel)**, according to WHO Global Health Estimates. The color bar represents the age-standardized suicide rate per 100,000 inhabitants.

To assess trends over time, we first examined changes in suicide rates between 2010 and 2019 across the European Union. According to WHO data, the rate of suicide has decreased from 2010 to 2019 in all but four EU Member States: Sweden, Netherlands, Spain, and Greece reported higher rates of suicide as opposed to those recorded in 2010 (Figure 1). A paired *t*-test confirmed a significant overall decrease in suicide rates between 2010 and 2019 (t = −4.75, df = 26, *p* < 0.001), with a mean reduction of 2.36 (95% CI [1.34–3.38]) suicides per 100,000 inhabitants. Additionally, correlation analysis revealed a strong positive correlation between suicide rates in 2010 and 2019 (r = 0.93, t = 13.14, df = 25, *p* < 0.001, 95% CI [0.86–0.97].

**Figure 2:**
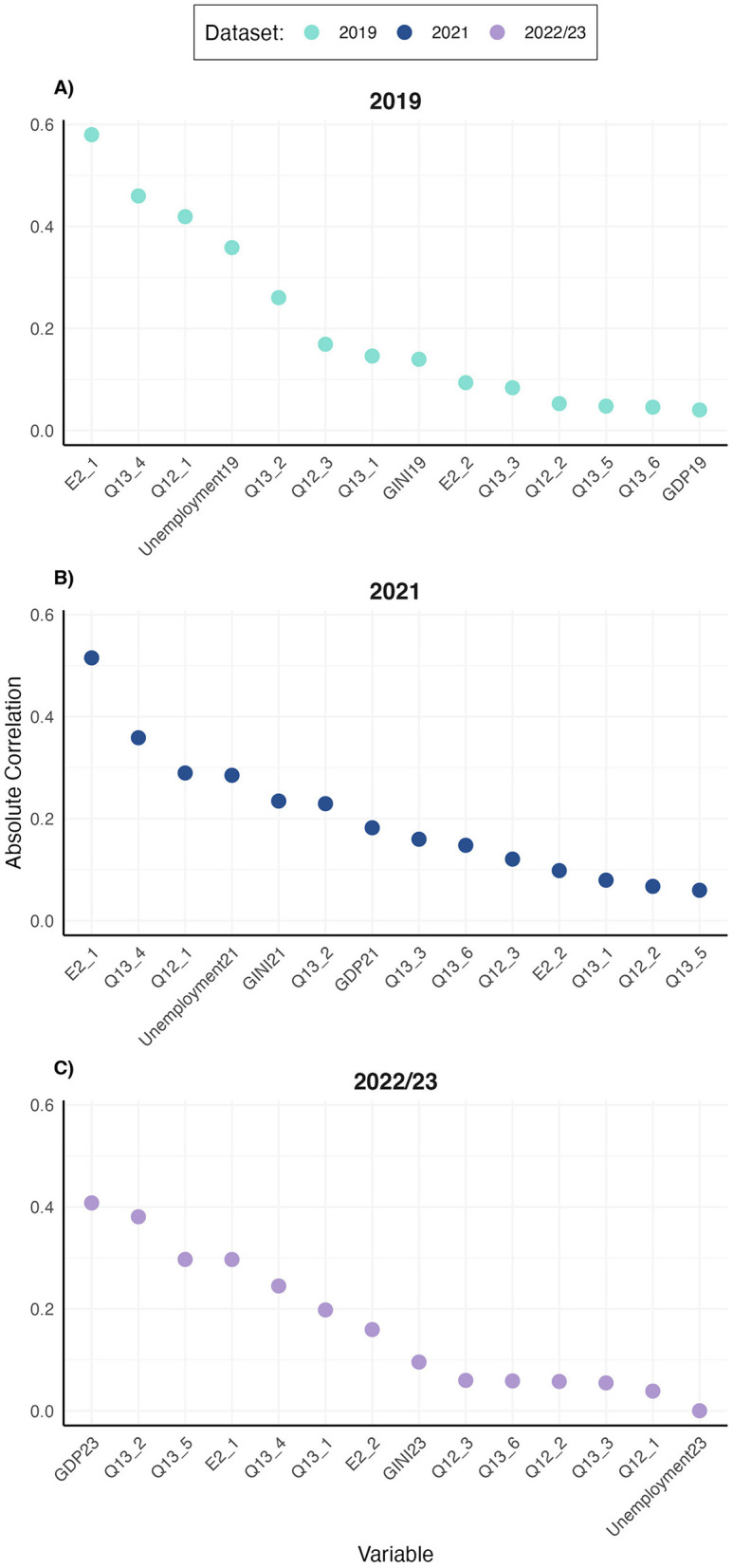
Correlations of stigma measures and socio-economic indicators with suicide rates across datasets. Absolute correlation values are plotted. **(A)** 2019 dataset with WHO suicide estimates. **(B)** 2021 dataset with suicide rates collected from Eurostat. **(C)** 2022/23 dataset with manually collected suicide rates.

Next, correlations between suicide rates and stigma measures and socio-economic factors were computed separately for each dataset. For the 2019 dataset, the most strongly correlated variables were E2_1 (“Disclosing a mental health condition would have a negative impact on my career,” r −0.58), Q13_4 (“People with mental health issues are seen as less sociable,” r = −0.46), Q12_1 (“Do you think that mental health patients are judged differently than other patients by society in general?” r = −0.42), and Unemployment rate (r = −0.36). In the 2021 dataset, the strongest correlations were observed for E2_1 (r = −0.52), Q13_4 (r = −0.36), Q12_1 (r = −0.29), and Unemployment rate (r = −0.29), mirroring the results obtained in the 2019 dataset. Within the 2022/23 dataset, the variable most strongly associated with suicide rates was GDP23 (r = −0.41), followed by stigma-related variables Q13_2 (“Mental health promotion is as important as physical health promotion,” r = 0.38) and Q13_5 (“People with mental health issues get less opportunities at work, in finding housing, in social activities etc.,” r = 0.30), as well as E2_1 (r = −0.30). The ranked correlation values for each dataset are visualized in Figure 2. These results informed the selection of variables for subsequent regression modeling.

Based on the correlation findings, we proceeded with hierarchical linear regression analyses for each dataset to examine the predictive value of stigma measures and socio-economic factors on suicide rates. The analysis of the 2019 dataset revealed a significant negative association between the stigma measure E2_1 and suicide rates (b = −0.201, *p* = 0.002). In the second model, which included both E2_1 and Q13_4, E2_1 remained significant (b = −0.162, *p* = 0.018), while Q13_4 did not demonstrate a statistically significant relationship (*p* = 0.220). The ANOVA comparison between Model 1 and Model 2 indicated no significant improvement in model fit (F = 1.588, *p* = 0.220). In the third model, which added Q12_1, E2_1 continued to be significant (b = −0.151, *p* = 0.044), but the ANOVA comparison with Model 2 showed no significant enhancement (F = 0.148, *p* = 0.704). The final model incorporated unemployment rates as a socio-economic factor; however, this addition did not result in a statistically significant improvement in model fit compared to Model 3 (F = 0.203, *p* = 0.657). E2_1 was no longer a significant predictor in the fourth model (b = −0.134, *p* = 0.116) (Table 1).

**Table 1:** Hierarchical linear regression models predicting 2019 suicide rates in EU member states.

**Independent variables**	**Model 1**				**Model 2**				**Model 3**				**Model 4**			
	**b**	**SE**	***t***	***p***	**b**	**SE**	***t***	***p***	**b**	**SE**	***t***	***p***	**b**	**SE**	***t***	***p***
(Intercept)	19.076	2.743	6.954	**< 0.001**	26.196	6.267	4.180	**< 0.001**	28.224	8.281	3.408	**0.002**	28.391	8.436	3.365	**0.003**
E2_1	−0.201	0.056	−3.560	**0.002**	−0.162	0.064	−2.532	**0.018**	−0.151	0.071	−2.128	**0.044**	−0.134	0.082	−1.638	0.116
Q13_4					−0.133	0.105	−1.260	0.220	−0.119	0.113	−1.053	0.303	−0.116	0.115	−1.004	0.326
Q12_1									−0.045	0.117	−0.384	0.704	−0.052	0.120	−0.434	0.668
Unemployment													−0.104	0.231	−0.451	0.657

E2_1: “Disclosing a mental health condition would have a negative impact on my career.” (rate of agreement), Q13_4: “People with mental health issues are seen as less sociable.” (rate of agreement), Q12_1: “Do you think that mental health patients are judged differently than other patients by society in general?” (rate of agreement), Unemployment: number of unemployed persons in 2019 as a percentage of the labor force. Values marked in bold represent statistically significant results (*p* < 0.05).

The examination of the 2021 dataset revealed a significant negative relationship between the stigma measure E2_1 and suicide rates (b = −0.192, *p* = 0.006). Upon inclusion of Q13_4 in the second model, E2_1 retained its significance (b = −0.166, *p* = 0.034), while Q13_4 did not demonstrate a statistically significant association (*p* = 0.484). The ANOVA comparison between Model 1 and Model 2 indicated no significant improvement in model fit (F = 0.507, *p* = 0.484). When Q12_1 was added in the third model, E2_1 remained significant (b = −0.172, *p* = 0.048); however, the ANOVA results showed no significant enhancement from Model 2 to Model 3 (F = 0.027, *p* = 0.870). The final model incorporated unemployment rates, but this did not lead to a significant improvement in overall model fit in ANOVA (F = 0.206, *p* = 0.654), As with the 2019 dataset, the predictive value of E2_1 diminished in Model 4, but remained marginally significant (b = −0.156, *p* = 0.098) (Table 2).

**Table 2:** Hierarchical linear regression models predicting 2021 suicide rates in EU member states.

**Independent variables**	**Model 1**				**Model 2**				**Model 3**				**Model 4**			
	**b**	**SE**	***t***	***p***	**b**	**SE**	***t***	***p***	**b**	**SE**	***t***	***p***	**b**	**SE**	***t***	***p***
(Intercept)	19.852	3.095	6.414	**< 0.001**	24.487	7.225	3.389	**0.002**	23.480	9.572	2.453	**0.022**	24.281	9.900	2.453	**0.023**
E2_1	−0.192	0.064	−3.008	**0.006**	−0.166	0.074	−2.254	**0.034**	−0.172	0.082	−2.091	**0.048**	−0.156	0.091	−1.719	0.100
Q13_4					−0.086	0.121	−0.712	0.484	−0.093	0.131	−0.714	0.483	−0.084	0.135	−0.622	0.540
Q12_1									0.022	0.135	0.165	0.870	0.005	0.142	0.037	0.971
Unemployment													−0.133	0.292	−0.454	0.654

E2_1: “Disclosing a mental health condition would have a negative impact on my career.” (rate of agreement), Q13_4: “People with mental health issues are seen as less sociable.” (rate of agreement), Q12_1: “Do you think that mental health patients are judged differently than other patients by society in general?” (rate of agreement), Unemployment: number of unemployed persons in 2021 as a percentage of the labor force. Values marked in bold represent statistically significant results (*p* < 0.05).

In the 2022/23 dataset, Model 1 identified a negative association between GDP and suicide rates (b = −0.00005), which approached statistical significance (*p* = 0.067). In the second model, which included both GDP and Q13_2, GDP remained marginally significant (b = −0.00005, *p* = 0.069), while Q13_2 showed a positive marginally significant relationship (b = 0.548, *p* = 0.091). The ANOVA comparison revealed a marginally significant improvement in model fit (F = 3.191, *p* = 0.091) for Model 2. In Model 3, GDP remained marginally significant (b = −0.00006, *p* = 0.087) whereas the added Q13_5 variable and the Q13_2 variable did not reach statistical significance (*p* = 0.728 and *p* = 0.113, respectively). The ANOVA comparison with Model 2 indicated no significant enhancement (F = 0.125, *p* = 0.729). In the fourth model, which included E2_1 along with GDP, Q13_2, and Q13_5, none of the predictors were statistically significant (GDP b = −0.00004, *p* = 0.277; Q13_2 b = 0.321, *p* = 0.463; Q13_5 b = 0.161, *p* = 0.556; E2_1 b = −0.121, *p* = 0.214), with an overall ANOVA comparison showing no significant improvement in model fit (F = 1.672, *p* = 0.214) (Table 3).

**Table 3:** Hierarchical linear regression models predicting 2022/23 suicide rates in EU member states.

**Independent variables**	**Model 1**				**Model 2**				**Model 3**				**Model 4**			
	**b**	**SE**	***t***	***p***	**b**	**SE**	***t***	***p***	**b**	**SE**	***t***	***p***	**b**	**SE**	***t***	***p***
(Intercept)	14.146	1.367	10.348	**< 0.001**	−35.559	27.855	−1.277	0.218	−35.991	28.584	−1.259	0.225	−22.604	29.886	−0.756	0.460
GDP (2023)	< 0.001	< 0.001	−1.947	0.066	< 0.001	< 0.001	−1.933	0.069	< 0.001	< 0.001	−1.815	0.087	< 0.001	< 0.001	−1.124	0.277
Q13_2					0.548	0.307	1.786	0.091	0.616	0.368	1.671	0.113	0.321	0.427	0.753	0.463
Q13_5									−0.071	0.201	−0.353	0.729	0.161	0.267	0.602	0.556
E2_1													−0.121	0.093	−1.293	0.214

GDP, gross domestic product per capita, Q13_2: “Mental health promotion is as important as physical health promotion.” (rate of agreement), Q13_5: “People with mental health issues get less opportunities at work, in finding housing, in social activities etc.” (rate of agreement), E2_1: “Disclosing a mental health condition would have a negative impact on my career.” (rate of agreement). Values marked in bold represent statistically significant results (*p* < 0.05).

## 4 Discussion

This study aimed to investigate the relationship between mental health stigma and suicide rates across EU Member States, expanding on prior work by Schomerus et al. (11). Our results indicate a significant overall decrease in suicide rates across the European Union between 2010 and 2019, with only four countries (Sweden, Netherlands, Spain, and Greece) reporting higher rates in 2019 compared to 2010. This general downward trend is encouraging and may reflect the effectiveness of suicide prevention strategies implemented during this period (12). However, the strong positive correlation between suicide rates in 2010 and 2019 suggests that countries with historically higher suicide rates tend to maintain relatively higher rates over time, highlighting the persistent nature of this public health challenge.

Correlation analysis revealed consistent associations between certain measures and suicide rates across the 2019 and 2021 datasets, with E2_1, Q13_4, Q12_1, and unemployment rates producing highest absolute correlation values. Due to methodological limitations in the 2022/23 dataset, including missing data points and variability of sources, we refrain from drawing definitive conclusions based on this time-point. A more accurate assessment of these relationships may be conducted once cross-national suicide data for 2023 are curated and made public, which would allow us to observe whether the trends observed in 2019 and 2021 persist, which seems likely given the high correlation between suicide rates over time.

Notably, the perceived career impact of disclosing mental health conditions (E2_1) emerged as a significant predictor of suicide rates in 2019 and 2021, even when controlling for other stigma-related variables. While E2_1 lost significance when unemployment was included in the model, ANOVA comparisons in both datasets indicated that neither the addition of unemployment nor other stigma variables significantly improved model fit. Interestingly, this association was negative, indicating that in countries where more people agreed that disclosing a mental health condition would have a negative impact on their career, lower suicide rates were reported. The unexpected finding that higher levels of perceived stigmatization correlate with lower suicide rates may initially seem paradoxical. However, there are several potential explanations for this relationship that warrant exploration.

One possibility is the role of awareness and precautionary behavior. It is conceivable that higher perceived stigma leads to greater vigilance among individuals with mental health issues. This increased awareness might prompt them to seek alternative coping mechanisms or engage in more intensive support networks as a way to mitigate the impact of potential discrimination. Supporting this idea, a recent study found that increased suicide stigma was correlated with an increased willingness to seek help from family and friends, and lower odds of experiencing current suicidality (7). While this study focused on suicide stigma rather than mental health stigma, its findings support the notion that stigma may drive individuals to seek support resources, consistent with the patterns observed in our results. In addition, societal awareness of stigmatization may lead to positive outcomes, such as improved peer support and community-based initiatives that ultimately contribute to reducing suicide risk. Indeed, Batterham et al. (23) found that higher exposure to suicide was related to higher levels of suicide literacy, and was not related to stigmatizing attitudes.

While awareness and precautionary behavior likely mediate the relationship between stigma and suicide, a crucial challenge in disentangling this connection lies in appropriate definition and accurate measurement of stigmatization. It has long been established that stigma is a complex multi-dimensional construct, which has historically suffered from a lack of clear definition and operationalization (24, 25). Highlighting the extent of this issue, an extensive review by Fox et. al has classified over 400 distinct measures of stigma used within scientific literature in the past decade, almost two thirds of which were created uniquely for the purposes of their respective research projects, and were not psychometrically validated (6).

Of note, since the publication of the Eurobarometer survey in 2010, no other international data have been collected using an identical measure of stigmatization, thus disabling a direct comparison of our results with the original work of Schomerus et al. (11). The 2010 Eurobarometer survey used a more generalized measure of social acceptance—whether respondents would feel comfortable talking to someone with a significant mental health problem (26). In contrast, the surveys published in 2022 and 2023 utilized more specific stigma indicators, such as perceptions about social judgment (Q12_1), perceived sociability (Q13_4), and career implications (E2_1) (13, 14).

To address this complexity of stigma definition and measurement, the Mental Illness Stigma Framework (MISF) has been developed to provide a theoretical basis for organizing research on the mechanisms through which individuals experience mental illness stigma (6). The framework differentiates between mechanisms pertaining to the perspective of the stigmatizer (stereotypes, prejudice, and discrimination), and those concerning the perspective of the stigmatized (experienced, anticipated, and internalized stigma). A third construct, termed perceived stigma, pertains to perceptions of societal beliefs, feelings, and behaviors toward people with mental illness, and can be shared both by those with and without mental illness. When applying the MISF framework to the items relevant to the current article, we can see that the 2010 stigma measure was most likely capturing the perspective of the stigmatizer (by assessing the level of prejudice), whereas the items from the Mental Health Eurobarometer were capturing perceived stigma. Importantly, the E2_1 measure seems to tap into a form of internalized stigma, which represents the application of negative stereotypes and prejudice to the self (6).

As pointed out by the authors of the MISF, it is relevant not to conflate the various stigma mechanisms, as they may be differentially related to outcomes of interest. For example, an extensive meta-analysis revealed that internalized stigma was a significant predictor of help-seeking, while perceived, experienced, and anticipated stigma showed no significant correlation (27). Similarly, a study by Mojtabai (28) found different stigmatizing beliefs to be differentially associated with individuals' willingness to seek help for a mental health condition. Specifically, viewing the those afflicted with mental health issues as dangerous or unlikely to recover correlated with increased help-seeking, while perceiving them as unpredictable or responsible for their condition was associated with reduced willingness to seek professional help. These differences highlight the complex nature of stigma, and the need to carefully select and evaluate specific measures of stigmatization when relating them to suicidal behavior.

### 4.1 Limitations

Several limitations of this study should be noted. First, the reliance on self-reported data for stigma measures may introduce bias, as respondents may underreport stigmatizing attitudes due to social desirability. Additionally, the fact that stigmatization is not measured systematically on an international scale limits our ability to reliably contrast our findings with those found by Schomerus et al. (11). Our chosen stigma indicators were a small segment of larger surveys assessing both mental and physical health. While we screened all items and included those evaluated to be indices of stigmatization, the nature of the datasets did not allow for an objective categorization of items assessing stigma, which may have limited the scope of our conclusions. To monitor both the changes in stigmatizing attitudes as well as their potential relation to national suicide rates, metrics of mental health stigmatization should be included in future iterations of both European and global public health surveys. Within this, careful attention should be given to the specific mechanisms of stigma that are being captured, allowing for a more structured delineation of the complex relationship between stigma and suicide. Finally, while reports indicate that between 60% and 98% of all suicide cases are linked to psychiatric disorders (29–31), there are cases in which suicide may not be attributable to mental illness. Due to the nature of the datasets used, the present study could not differentiate between cases related or unrelated to mental health conditions. It may be beneficial for future work to explore the relationship between stigma and suicide rates with a more nuanced understanding of the causes behind suicides, including non-mental illness-related factors.

## 5 Conclusion

Both suicidal behavior and the stigma surrounding mental health continue to be pressing issues in today's society. Despite decreasing rates of suicide and a rise in stigma prevention efforts, the persistence of these issues highlights the need for continued attention and intervention. This article highlights the multifaceted nature of the relationship between stigma and suicide rates, considering potential protective factors arising from perceived stigmatization. Whereas some associations have been demonstrated for the impact of perceived and internalized stigma on the risk of suicidal behavior (32–35), public stigma seems to exhibit an even more complex interaction with suicide rates. While the findings challenge some of the conventional wisdom by showing negative correlations between stigma and suicide rates, they also underscore the need for more nuanced and longitudinal research approaches. Future research should delve deeper into these dynamics, taking into account cultural, policy, and individual factors that modulate the apparent protective effect of stigma awareness on suicide outcomes. The present work adds to the body of literature calling for a comprehensive and systematical assessment of different facets of stigma on a societal level, given the gravity of outcomes related to the experience of stigmatization.

## Data Availability

The original contributions presented in the study are included in the article/supplementary material, further inquiries can be directed to the corresponding author.
